# *Aspergillus oryzae* Fermentation Extract Alleviates Inflammation in *Mycoplasma pneumoniae* Pneumonia

**DOI:** 10.3390/molecules28031127

**Published:** 2023-01-23

**Authors:** Hui-Yu Lee, Chun-Chia Chen, Chia-Chen Pi, Chun-Jen Chen

**Affiliations:** 1 Department of Biochemical Science and Technology, National Taiwan University, Taipei 10617, Taiwan; 2 King’s Ground Biotech Co., Ltd., Pintung 91252, Taiwan

**Keywords:** *Aspergillus oryzae*, *Mycoplasma pneumoniae*, pneumonia, anti-inflammation

## Abstract

The filamentous fungus *Aspergillus oryzae*, also known as koji mold, has been used for centuries in the production of fermented foods in East Asia. *A. oryzae* fermentation can produce enzymes and metabolites with various bioactivities. In this study, we investigated whether *A. oryzae* fermentation extract (AOFE) has any effect on *Mycoplasma pneumoniae* (Mp) pneumonia. We performed solid-state fermentation of *A. oryzae* and obtained the ethanol extract. AOFE was analyzed by HPLC, and the major component was identified to be kojic acid. In vitro, AOFE suppressed Mp growth and invasion into A549 lung epithelial cells as determined by the gentamicin protection assay. AOFE treatment also suppressed Mp-stimulated production of tumor necrosis factor (TNF)-α and interleukin (IL)-6 at mRNA and protein levels in murine MH-S alveolar macrophages. In a mouse model of Mp pneumonia, Mp infection induced a marked pulmonary infiltration of neutrophils, which was significantly reduced in mice pre-treated orally with AOFE. AOFE administration also suppressed the production of proinflammatory cytokines and chemokines in the lungs. Collectively, our results show that AOFE has the potential to be developed into a preventive/therapeutic agent for Mp pneumonia.

## 1. Introduction

*Mycoplasma pneumoniae* (Mp) is a common cause of community-acquired pneumonia, especially in children and adolescents, and it can cause both upper and lower respiratory infections [1]. Mp pneumonia is usually self-limiting, but life-threatening refractory pneumonia may occur sometimes [2]. The pathogenesis of Mp infection is complicated, and several mechanisms are involved, including invasive damage, adhesion damage, toxic damage, membrane fusion damage, nutrition depletion, and inflammatory damage [3]. During Mp infection, Mp first attaches to the bronchial epithelial cells, then activates alveolar macrophages, which are the major innate immune cells in the lungs. Animal studies have shown that the Toll-like receptor 2 (TLR2)-myeloid differentiation primary response protein 88 (MyD88)-nuclear factor kappa B (NF-κB)- signaling pathway plays a major role in Mp-stimulated activation of alveolar macrophages and bronchial epithelial cells [4,5,6]. Upon activation, alveolar macrophages can produce a range of proinflammatory cytokines and chemokines, which initiate an inflammatory response [7]. The major pathological feature of human Mp pneumonia is the accumulation of neutrophils and lymphocytes in the alveolar spaces, with marked infiltration of plasma cells in the peri-bronchovascular areas [8]. Neutrophils are the major inflammatory cells recruited during the early stage of Mp pneumonia. Although neutrophils are phagocytic cells, they are not essential for the elimination of Mp from the lungs, and in contrast, neutrophils were shown to contribute to lung injury in Mp pneumonia [9,10]. Therefore, immunomodulatory agents that can inhibit neutrophil infiltration are likely to suppress the exacerbation of Mp pneumonia [11].

The filamentous fungus *Aspergillus oryzae*, also known as koji mold, has been used for centuries in the production of fermented foods, such as rice wine (sake), soy sauce, and soybean paste (miso) in East Asia [12,13]. *A. oryzae* is generally recognized as safe (GRAS) by the Food and Drug Administration (FDA) and therefore is used for the production of various enzymes, including amylases, proteases, and glutaminases [14]. *A. oryzae* is also known to produce a variety of bioactive metabolites, such as angiotensin I-converting enzyme inhibitor peptides, biotin, Bifidobacterium-stimulating peptides, beta-glucan, citric acid, deferriferrichrysin, 14-dehydroergosterol, ergothioneine, ethyl-α-D-glucoside, ferulic acid, glycosylceramide, kojic acid, oligosaccharides, polyamines, pyroglutamyl leucine, pyranonigrin A, and resistant proteins [15]. Among the metabolites of *A. oryzae*, kojic acid and its derivatives have been shown to exhibit a variety of biological functions [16], including antimicrobial [17], antioxidant [18], anti-inflammatory [19], and wound-healing [20] activities. 

Studies have shown that *A. oryzae* fermentation products have anti-inflammatory effects [15,21,22], and we hypothesize that *A. oryzae* fermentation extract (AOFE) may attenuate Mp-induced pneumonia. In this study, we tested this hypothesis and found that AOFE suppressed Mp-induced inflammation in alveolar macrophage and alleviated Mp pneumonia in mice.

## 2. Results

### 2.1. High-Performance Liquid Chromatography (HPLC) Profile of AOFE

**Figure 1:**
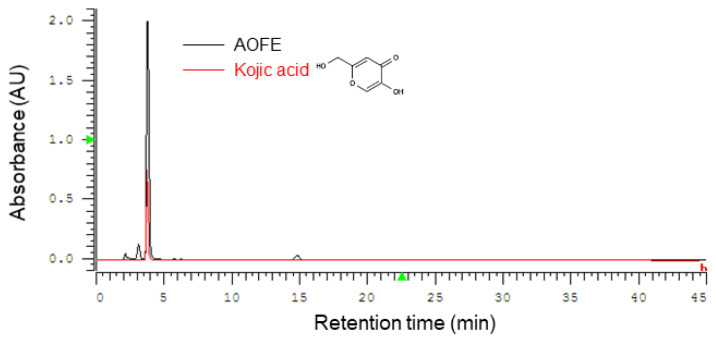
Representative HPLC chromatograms of AOFE and kojic acid standard. Kojic acid was found to be the major component of AOFE.

*A. oryzae* culture was performed by solid-state fermentation at 30 °C for 4 days, and the fermentation product was extracted with 70% ethanol to obtain AOFE. A representative HPLC chromatogram of AOFE is shown in Figure 1. Kojic acid [5-hydroxy-2-(hydroxymethyl)-4H-pyran-4-one] was found to be the major compound in AOFE, and the concentration of kojic acid was 16.5 mg/g AOFE.

### 2.2. AOFE Inhibits the Growth of Mp

**Figure 2:**
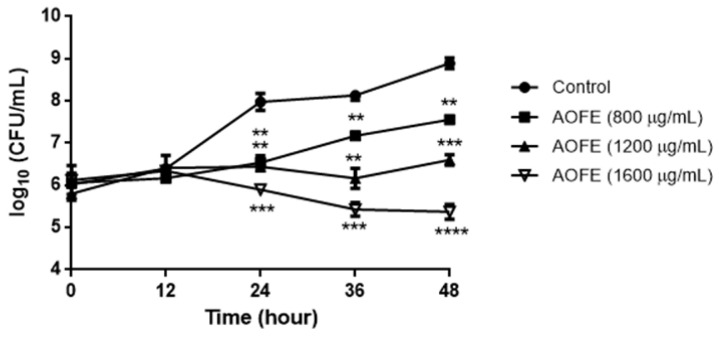
Effect of AOFE on Mp growth. Mp was cultured without (control) or with different concentrations of AOFE. The cultures were sampled at 0, 12, 24, 36, and 48 h and quantified for the titers of Mp by qPCR. The results shown are representative of two independent experiments with similar results (*n* = 3). ** *p* < 0.01, *** *p* < 0.001, **** *p* < 0.0001 versus the control.

To determine whether AOFE has any effect on Mp growth, Mp was cultured in the presence of AOFE at different concentrations for 48 h, and Mp titers were determined by quantitative PCR (qPCR). As shown in Figure 2, AOFE significantly inhibited Mp growth in a dose-dependent manner. At 48 h, the inhibitory rates of Mp growth by AOFE at 800, 1200, and 1600 mg/mL were 95.37%, 99.5%, and 99.97%, respectively. 

### 2.3. AOFE Reduces the Invasion of Mp into Lung Epithelial Cells

**Figure 3:**
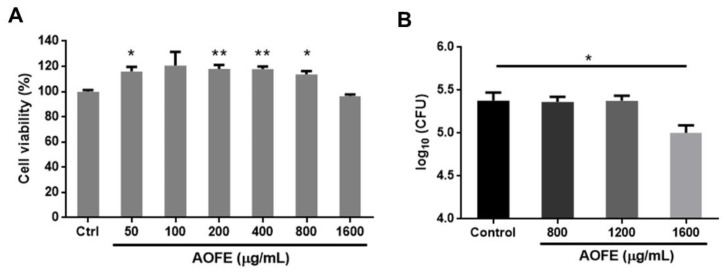
Effect of AOFE on the invasion of Mp in A549 cells. (**A**) A549 cells were treated with different concentrations of AOFE for 20 h, and cell viability was assessed by MTT assay. Untreated cells served as the control. The data shown are representative of three independent experiments (*n* = 3). (**B**) A549 cells were pre-treated with AOFE at indicated concentrations for 2 h, followed by Mp infection (MOI = 100) for 24 h. The amount of Mp internalized into A549 cells was determined by the gentamicin protection assay and qPCR, as described in the Materials and Methods. The results shown are representative of two independent experiments with similar results (*n* = 3). * *p* < 0.05, ** *p* < 0.01 versus the controls.

One of the mechanisms by which Mp causes damage to the host’s respiratory epithelium is by invading airway epithelial cells [23,24]. We next investigated whether AOFE had any effect on the invasion by Mp of human lung carcinoma A549 cells by using the gentamicin protection assay. We first examined the effect of AOFE on the viability of A549 cells. As shown in Figure 3A, AOFE at 50–1600 μg/mL had no significant adverse effect on cell viability. To determine whether AOFE affected the internalization of Mp, A549 cells were infected with Mp in the presence or absence of different concentrations of AOFE for 24 h. After killing extracellular Mp with gentamicin, the internalized Mp was quantified by qPCR. As shown in Figure 3B, in the presence of 1600 μg/mL of AOFE, the level of Mp internalized into A549 cells was significantly reduced to 42.13% of the control. 

### 2.4. AOFE Reduces Mp-Induced Inflammatory Response in Alveolar Macrophages

**Figure 4:**
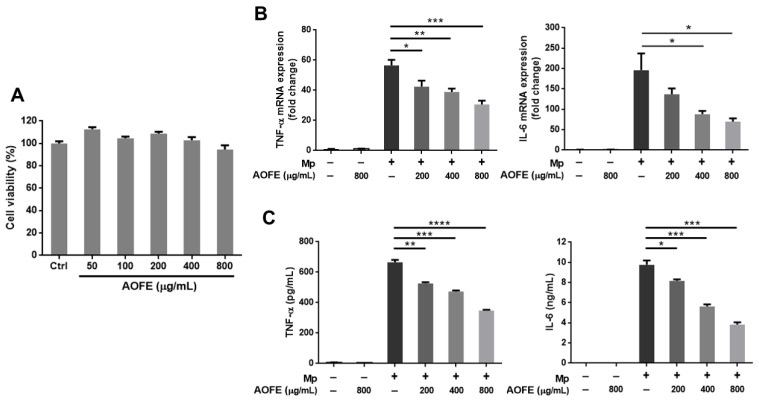
AOFE inhibits Mp-stimulated inflammatory response in MH-S cells. (**A**) MH-S cells were treated with different concentrations of AOFE for 20 h, and cell viability was assessed by MTT assay. Untreated cells served as the control. (**B**,**C**) MH-S cells were pre-treated with or without AOFE for 2 h, and the cells were stimulated with Mp (MOI = 100) for 4 h (**B**) or 20 h (**C**). Cells left untreated or treated with AOFE alone served as controls. At 4 h after Mp stimulation, total RNA was extracted from MH-S cells and analyzed for TNF-α and IL-6 mRNA expression by RT-qPCR (**B**). At 20 h after Mp stimulation, TNF-α and IL-6 levels in the culture fluids were determined by ELISA (**C**). All data shown are representative of two independent experiments with similar results (*n* = 3). * *p* < 0.05, ** *p* < 0.01, *** *p* < 0.001, **** *p* < 0.0001.

The inflammatory responses induced by Mp infection appear to contribute to pathological damage in the lungs [3], and immunosuppression was shown to reduce lung injury caused by Mp infection [11]. To investigate whether AOFE may modulate host immune responses and reduce excessive inflammation, we first examined the effect of AOFE on Mp-induced inflammation in alveolar macrophages, which are the major innate immune cells in the lung. Murine alveolar macrophage MH-S cells were stimulated with Mp in the presence or absence of AOFE. As shown in Figure 4A, the viability of MH-S cells was not significantly affected after incubation with 50–800 μg/mL of AOFE for 20 h. Therefore, 800 μg/mL was used as the maximum dose when examining the effect of AOFE on Mp-induced proinflammatory cytokine production in MH-S cells. Results showed that AOFE dose-dependently reduced the expression of tumor necrosis factor (TNF)-α and interleukin (IL)-6 at mRNA (Figure 4B) and protein (Figure 4C) levels, indicating that AOFE could suppress Mp-stimulated activation of MH-S cells. In the presence of 800 μg/mL of AOFE, the levels of TNF-α and IL-6 in the culture medium were reduced by 47.62% and 60.76%, respectively.

### 2.5. AOFE Reduces Mp-Induced Lung Inflammation in Mice

**Figure 5:**
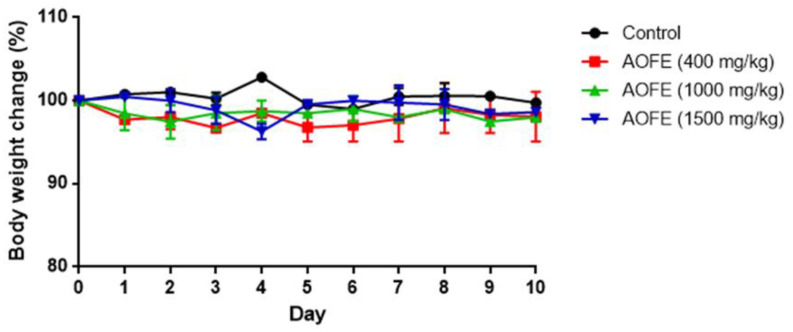
Effect of AOFE administration on mouse body weight. BALB/c mice were administered p.o. with 100 μL of 50% ethanol (control) or AOFE (400, 1000, or 1500 mg/kg bw in 50% ethanol) daily for 10 days. Mouse body weight was measured daily and compared with the weight on day 0 (*n* = 3).

We next investigated whether AOFE could reduce lung inflammation in Mp-challenged mice. Neutrophil infiltration is generally recognized as one of the features of Mp pneumonia. While neutrophils are not essential for the clearance of Mp from the lungs [9,10], in the situation of “hyperinflammation,” excessive accumulation of neutrophils might cause lung injury by releasing myeloperoxidase (MPO) and proteases [10,25]. To investigate whether AOFE reduces inflammation in Mp pneumonia, we first examined if the administration of AOFE had an adverse effect on mouse health by feeding mice daily with different doses of AOFE for 10 days. As shown in Figure 5, oral administration of AOFE at 400, 1000, and 1500 mg/kg body weight (bw)/day did not have any significant effect on mouse body weight. 

**Figure 6:**
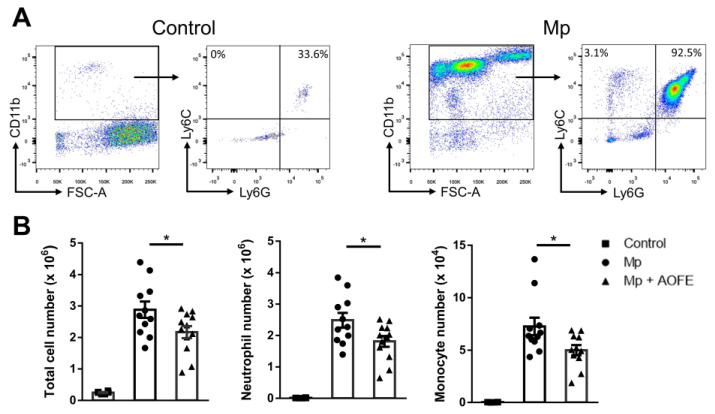
AOFE administration attenuates inflammatory cell infiltration in Mp-challenged mice. BALB/c mice were treated p.o. with vehicle (control and Mp groups) or 1500 mg/kg bw of AOFE (Mp + AOFE group) daily for 7 days before Mp inoculation. Mice in the Mp and Mp + AOFE groups were inoculated i.n. with Mp, and mice in the control group were treated with the vehicle. At 24 h post Mp inoculation, BALF cells were analyzed for surface expression of CD11b, Ly-6C, and Ly-6G by flow cytometry. (**A**) Representative density plots and gating strategies of BALF cells in the control and Mp groups. CD11b^+^Ly6G^+^Ly6C^+^ cells represent neutrophils, and CD11b^+^Ly6G^−^Ly6C^+^ cells represent monocytes. (**B**) Neutrophil and monocyte numbers in BALF were determined by multiplying the total cell numbers by the percentage of each cell population. The data shown are combined from 3 independent experiments. * *p* < 0.05.

We then chose the dose of 1500 mg/kg bw to evaluate the effect of AOFE on Mp pneumonia. Mice were administered with AOFE or vehicle control (50% ethanol) daily for 7 days before intranasal (i.n.) inoculation with Mp. At 24 h after Mp challenge, mice were sacrificed, and bronchoalveolar lavage fluid (BALF) was analyzed for inflammatory cell infiltration and cytokine production. Infection of Mp resulted in a marked influx of CD11b^+^ inflammatory cells into the lungs, and the recruited cells were primarily CD11b^+^Ly6G^+^Ly6C^+^ neutrophils and a small percentage of CD11b^+^Ly6G^−^Ly6C^+^ monocytes (Figure 6A). AOFE treatment significantly reduced the infiltration of neutrophils and monocytes into the lungs (Figure 6B). 

**Figure 7:**
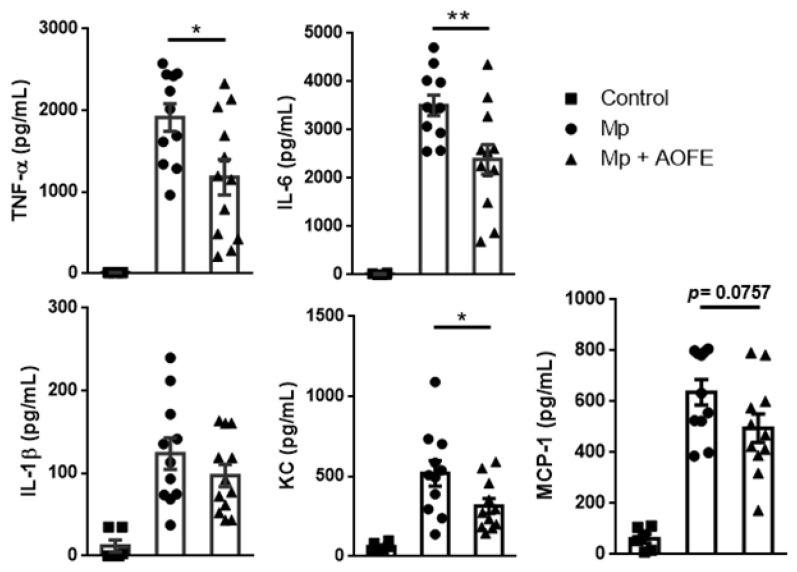
AOFE administration reduces proinflammatory cytokine production in Mp-challenged mice. BALB/c mice were treated as described in Figure 6. At 24 h post Mp inoculation, TNF-α, IL-6, IL-1β, KC, and MCP-1 levels in BALF were analyzed by ELISA. The data shown are combined from 3 independent experiments. * *p* < 0.05, ** *p* < 0.01.

We next measured the levels of proinflammatory cytokines (TNF-α, IL-6, and IL-1β) and chemokines for neutrophils (KC) and monocytes (MCP-1) in the BALF. Mice challenged with Mp produced high amounts of TNF-α and IL-6, and in contrast, levels of these cytokines were significantly reduced in AOFE-treated animals (Figure 7). Mp challenge also induced the production of KC and MCP-1, which were also markedly reduced by AOFE treatment. Together, these results showed that AOFE treatment could effectively reduce Mp pneumonia in mice. 

## 3. Discussion

In the present study, we found that AOFE, the ethanol extract of *A. oryzae* fermentation product, inhibited the growth and cell invasion of Mp. Most importantly, AOFE suppressed Mp-induced alveolar macrophage activation and alleviated Mp pneumonia in a mouse model. To our knowledge, this is the first report showing that *A. oryzae* fermentation produces substances that attenuate Mp pneumonia. 

Our data showed that AOFE effectively inhibited the growth of Mp. HPLC analysis of AOFE showed that kojic acid was the major component in the extract, and therefore kojic acid could be the active compound inhibiting Mp growth. Wu et al. reported that kojic acid had antibacterial and anti-biofilm properties against Gram-positive bacteria (*Listeria monocytogenes*, *Bacillus subtilis*, and *Staphylococcus aureus*) and Gram-negative bacteria (*Escherichia coli* and *Salmonella typhimurium*). The mechanism of bacterial killing by kojic acid was primarily via damaging the cell membrane integrity [17]. Whether and how kojic acid may inhibit Mp growth require further investigations. Currently, macrolides and tetracyclines are the major antibiotics for treating Mp infections; however, resistance to macrolides has been a worldwide concern in recent years [26,27]. Our data suggest that AOFE has the potential to be used as a novel agent against Mp infections.

Mp has typically been considered an extracellular pathogen, and the adherence of bacteria to the host airway epithelium is required for the colonization and induction of disease [28,29]. In addition to adhering to the surface of epithelial cells, Mp has been shown to be able to invade lung epithelial A549 cells and localize in the cytoplasm and perinuclear regions [23]. Mp infection in A549 cells also induces ROS generation and DNA damage [24]. Its internalization into lung epithelial cells may provide Mp with a privileged site where it could evade the actions of the immune system and antibiotics. Using the gentamicin protection assay, our data showed that a high dose (1600 μg/mL) of AOFE could reduce the invasion of Mp into A549 cells. It is possible that AOFE may interfere with the adhesion and/or invasion of Mp to the cells. However, since the internalization of Mp was measured at 24 h after infection, we could not exclude the possibility that the direct bactericidal effect of AOFE may also contribute to a reduction in Mp invasion into A549 cells. 

Our data showed that AOFE significantly suppressed Mp-induced production of proinflammatory cytokines in MH-S alveolar macrophages. It is possible that AOFE interferes with the TLR2-MyD88-NF-κB signaling pathway, which is known to be activated by Mp [4,5,6]. The inhibition of Mp-stimulated activation of alveolar macrophages by AOFE may account for the reduced Mp pneumonia in mice treated with AOFE. The induction of inflammation and recruitment of phagocytes are important for controlling bacterial pneumonia [30]. However, in Mp pneumonia, studies have shown that macrophages, but not neutrophils, play a critical role in the clearance of Mp from the lungs [9]. Clinical studies have shown that increased accumulations of neutrophils and lymphocytes are associated with the severity of Mp pneumonia, and may contribute to the pathogenesis [25,31]. In an animal model of Mp pneumonia, Tamiya et al. showed that depletion of neutrophils did not affect the number of Mp in the BALF, but significantly reduced tissue injury, indicating that neutrophils contribute to lung injury, but not Mp clearance [10]. Our data showed that AOFE administration significantly reduced the infiltration of neutrophils into the lungs, suggesting that lung injury may also be reduced. In addition, AOFE treatment markedly reduced the levels of proinflammatory cytokines (TNF-α, IL-6, and IL-1β) and chemokines (KC and MCP-1) in the BALF. Similarly to our observation, cytokine signatures associated with Mp pneumonia severity have been characterized to be TNF-α, IL-6, IL-1β, MCP-1 IL-4, IFN-γ, and IL-10 [32]. Based on current knowledge from clinical and animal studies, the host immune response is likely to play a pathological role in Mp pneumonia, and immunosuppressive agents such as AOFE may reduce lung injury caused by Mp infection [11,33,34].

In conclusion, our data demonstrate that AOFE, the ethanol extract of *A. oryzae* fermentation product, can inhibit Mp growth and invasion into lung epithelial cells, as well as alleviate Mp-stimulated inflammatory responses in alveolar macrophages and in mice. Although the animal model used in this study may not fully recapitulate the human condition, our data suggest that AOFE has the potential to be developed into a preventive/therapeutic agent for Mp pneumonia. 

## 4. Materials and Methods

### 4.1. Mp and Culture Conditions

*M. pneumoniae* (ATCC 29342) was purchased from American Type Culture Collection (ATCC, Manassas, VA, USA) and cultured at 37 °C with shaking (120 rpm) in the modified ATCC Medium 2611 (pH 7.4) containing 17% fetal bovine serum (FBS, Biological Industries, Beit-Haemek, Israel), 0.35% PPLO broth (STBIO Media, Taipei, Taiwan), 0.725% yeast extract (Neogen, Lansing, MI, USA), 1% pancreatic digest of casein (STBIO Media), 0.5% pancreatic digest of gelatin (Neogen), 0.5 X CMRL 1066 medium (HiMedia, Mumba, India), 0.5% D-glucose (BioShop Canada, Burlington, VT, Canada), and 0.002% phenol red (PeproTech, Rocky Hill, NJ, USA). To determine the colony-forming unit (CFU), 10-fold dilutions were prepared and 10 μL aliquots were plated on ATCC Medium 2611 agar and incubated at 37 °C in 5% CO_2_ for 7 days. Colonies were counted under a microscope. 

### 4.2. Cell Lines

Murine alveolar macrophage MH-S cells were cultured in RPMI 1640 (Thermal Scientific HyClone, Logan, UT, USA) supplemented with 10% FBS. Human lung carcinoma A549 cells were cultured in DMEM (Thermal Scientific HyClone) supplemented with 10% FBS. 

### 4.3. A. oryzae Fermentation and AOFE Preparation

*A. oryzae* FG003 was used for solid-state fermentation, and the substrate was a mixture of spent coffee grounds (60%), corn grits (30%), and rice husks (10%), which has a C/N ratio of 50 ± 3% and a moisture content of 65%. The substrate pH was 5.0–5.5. After autoclave sterilization, *A. oryzae* spore powders were added into the substrate (6.5 × 10^7^ spores/g substrate), and the fermentation was carried out in a 30 °C incubator for 4 days. Due to heat generation during fermentation, the culture was agitated intermittently to maintain the core temperature at 34–36 °C. The crude fermentation product was dried at 50 °C to reduce the moisture content (<7.5%), followed by grinding into powders. The powders were then extracted with 70% ethanol (1:5 *w*/*v*) and sonicated for 1 h. After centrifugation at 12,000× *g* for 10 min, the supernatant was concentrated using a rotary evaporator (Eyela, Japan) to obtain AOFE. The recovery of the dried AOFE was approximately 20–30% (*w*/*w*), and the dried AOFE was stored at −20 °C until use. When used in cell culture experiments, AOFE was dissolved in 50% ethanol (40 mg/mL) and passed through a 0.22-μm filter.

### 4.4. HPLC Analysis

The analysis of AOFE was performed using an HPLC system (Hitachi D2000, Tokyo, Japan), which consisted of an L-2130 HTA pump, an L-2200 autosampler, and an L-2455 photodiode array detector. All solvents used were HPLC grade, and all reagents were analytical grade. The extracted compounds of AOFE and the kojic acid standard (Sigma-Aldrich, St. Louis, MO, USA) were separated using an Inertsil ODS-2 C18 column (250 mm × 4.6 mm, 5 μm) with a gradient mobile phase of 0.1% formic acid in ddH_2_O (solvent A) and 0.1% formic acid in acetonitrile (solvent B). The analysis involved a gradient elution at a flow rate of 1 mL/min: 0–10 min, 10% B; 10–24 min, 10% B to 20% B; 24–30 min, 20% B to 22% B; 30–35 min, 22% B to 25% B; 35–35.1 min, 25% B to 10% B; 35.1–45 min, 10% B. Injection volume was 10 μL. The column temperature was maintained at 30 °C, and the absorbance of the eluate was monitored continuously at 280 nm. Chromatographic peaks were identified by spiking the samples with standard compounds, and their UV spectra and retention times were matched with standard compounds.

### 4.5. Extraction of Mp DNA

The extraction of Mp DNA was performed as previously described [35]. The Mp culture broth was centrifuged at 8000× *g* for 30 min, and the cell pellet was washed once with Dulbecco’s phosphate-buffered saline (DPBS) and resuspended in 50 μL of lysis buffer [10 mM Tris-HCl (pH 8.3), 50 mM KCl, 2 mM MgCl_2_, 0.001% gelatin, 0.5% NP-40, 0.5% Tween-20 and proteinase-K (100 μg/mL)]. The cell suspension was first incubated at 60 °C for 1 h and then at 95 °C for 10 min. After centrifugation at 16,000× *g* for 5 min, the supernatant containing Mp DNA was collected and stored at −20 °C until used.

### 4.6. Detection of Mp by qPCR

To quantify Mp titers (CFU/mL) by qPCR, Mp DNA was extracted, and the P1 adhesin gene was used as the target for amplification as previously described [35]. The primer sequences were 5′-AACCTCGCGCCTAATACTAATACG-3′ (forward) and 5′-TTGCGGCGTTGCTTTCAG-3′ (reverse). qPCR was performed using IQ SYBR Green Supermix (Bio-Rad, Hercules, CA, USA) on a Bio-Rad CFX Connect Real-Time PCR System. The PCR conditions were as follows: denaturation at 94 °C for 4 min, followed by 35 cycles of 95 °C for 45 s, 50 °C for 1 min, and 72 °C for 2 min, with a final extension at 72 °C for 5 min. DNA extracted from Mp with a known CFU was serially diluted and used for generating a standard curve by plotting the cycle threshold (Ct) against the logarithm of Mp quantity (log_10_ CFU equivalent). 

### 4.7. Cell Viability Analysis

MH-S cells (2 × 10^4^ cells/well) or A549 cells (1 × 10^4^ cells/well) were seeded in 96-well plates. After overnight culture, cells were treated with different concentrations of AOFE and incubated for 20 h. The medium was replaced with 200 μL of thiazolyl blue tetrazolium bromide (MTT, Alfa Aesar) at 0.5 mg/mL in the culture medium. After 4 h of incubation at 37 °C, the medium was removed, and the MTT-formazan crystals formed in metabolically active cells were dissolved in 200 μL of dimethyl sulfoxide. The absorbance at 570 nm was measured using a microplate reader (Multiskan FC, Thermo Scientific, Milford, MA, USA). 

### 4.8. In Vitro Stimulation of MH-S Cells with Mp

MH-S cells were seeded in 6-well plates (5 × 10^5^ cells/well), and after overnight culture, cells were pre-treated with different concentrations of AOFE for 2 h before the addition of Mp (MOI = 100). At 20 h after Mp treatment, TNF-α and IL-6 levels in the culture medium were determined by ELISA (BioLegend, San Diego, CA, USA). To determine mRNA expression, MH-S cells were treated with AOFE and Mp as described above, and at 4 h after Mp treatment, total RNA was extracted using 3-Zol (Trizol) (Cyrusbioscience, Taipei, Taiwan) and reverse-transcribed into cDNA with oligo (dT) primers (Deoxy+ HiSpec Reverse Transcriptase, Yeastern Biotech, Taipei, Taiwan). Reverse transcription-quantitative PCR (RT-qPCR) was performed using gene-specific primers and IQ SYBR Green Supermix (Bio-Rad) on a Bio-Rad CFX Connect Real-Time PCR System. The mouse TNF-α primer sequences were 5′-CCCACACCGTCAGCCGATTT-3′ (forward) and 5′-GTCTAAGTACTTGGGCAGATTGACC-3′ (reverse). The mouse IL-6 primer sequences were 5′-CTGATGCTGGTGACAACCAC-3′ (forward) and 5′-TCCACGATTTCCCAGAGAAC-3′ (reverse). The mouse β-actin primer sequences were 5′-AGGTGTGCACCTTTTATTGGTCTCAA-3′ (forward) and 5′-TGTATGAAGGTTTGGTCTCCCT-3′ (reverse). All RT-qPCR values of interested genes were normalized to β-actin as an internal control. All data were shown as fold-change relative to the untreated samples. 

### 4.9. Gentamicin Protection Assay

Internalization of Mp into A549 cells was determined by the gentamicin protection assay as previously described with modifications [23]. A549 cells (5 × 10^5^ cells/well) were seeded in 6-well plates, and after overnight culture, cells were pre-treated with different concentrations of AOFE for 2 h before the addition of Mp (MOI = 100). At 24 h after infection, cells were washed once with DPBS and incubated for an additional 2 h in DMEM containing 400 μg/mL gentamicin (Sigma-Aldrich). After washing three times with DPBS to remove the bacteria that were not internalized, the cells were lysed using 50 μL of the lysis buffer described above for DNA extraction, and the DNA was precipitated with 50 μL isopropanol. After centrifugation at 19,000× *g* for 30 min, the pellet was dissolved in ddH_2_O and the amount of Mp DNA was quantified by qPCR. 

### 4.10. Mp Growth Curve

Mp was grown in the modified ATCC Medium 2611 containing different concentrations of AOFE at 37 °C. The culture broth was collected at 0, 12, 24, 36, and 48 h, and Mp DNA was extracted and quantified by qPCR. 

### 4.11. Animals and Mp Inoculation

Female BALB/c mice (6 weeks old) were purchased from the National Laboratory Animal Center (Taipei, Taiwan) and housed in the animal biosafety level 2 facilities at the Animal Resource Center of National Taiwan University. All animal studies were approved by the Institutional Animal Care and Use Committee of National Taiwan University. To determine the effect of AOFE administration on mouse body weight, mice were administered orally (p.o.) with 100 μL of 50% ethanol or AOFE (400, 1000, or 1500 mg/kg bw in 50% ethanol) daily for 10 days, and animal body weight was measured. In the Mp challenge experiments, mice were divided into three groups: control, Mp, and Mp + AOFE. To investigate the effect of AOFE on Mp pneumonia, mice were treated p.o. with 100 μL of 50% ethanol (control and Mp groups) or 1500 mg/kg bw of AOFE in 50% ethanol (Mp + AOFE group) daily for 7 days before Mp inoculation. Mice in the Mp and Mp + AOFE groups were inoculated i.n. with 20 μL of Mp (5 × 10^8^ CFU in ATCC Medium 2611), and mice in the control group were treated i.n. with 20 μL of ATCC Medium 2611. At 24 h after Mp inoculation, mice were sacrificed, and BALF was harvested. 

### 4.12. Bronchoalveolar Lavage and BALF Analysis

Bronchoalveolar lavage was performed by instilling 0.5 mL of PBS containing 2% FBS into the trachea four times via a trachea cannula (Angiocatch^®^, BD Biosciences), and BALF was harvested by gentle aspiration. After centrifugation (350× *g*, 5 min), BALF supernatants were assayed for TNF-α, IL-6, IL-β, KC, and MCP-1 by ELISA (all from BioLegend). After treatment with ammonium-chloride-potassium buffer to lyse erythrocytes, BALF cells were counted and analyzed by flow cytometry.

### 4.13. Flow Cytometric Analysis

To analyze cell surface markers, single-cell suspensions were first incubated with mAb 2.4G2 for 30 min to block FcγRIIB/III receptors, followed by incubation with various mAbs (all from BioLegend) as indicated for 30 min at 4 °C. The following mAbs were used: CD11b-PE, Ly6G-FITC, and Ly6C-APC. Dead cells were excluded using 7-AAD staining. Samples were analyzed on a FACSCanto II flow cytometer (BD Biosciences), and data were analyzed using FlowJo software (BD Biosciences). 

### 4.14. Statistical Analysis

Statistical analysis was performed using an unpaired, two-tailed Student’s *t*-test, and *p* < 0.05 was considered statistically significant. Data are reported as mean ± SEM.

## Data Availability

The data presented in this study are available from the corresponding author upon reasonable request.

## Sample Availability

Samples of AOFE are available from the authors.
